# Case Report: Sequential high-intensity focused ultrasound and focused ultrasound for cervical and vaginal low-grade squamous intraepithelial lesions

**DOI:** 10.3389/fonc.2026.1845584

**Published:** 2026-09-14

**Authors:** Yini Wei, Ganggang Li, Yunhua Zhang, Yushan Li, Jingyi Zhang, Lei Li, Qiuling Shi, Ru Meng, Jinjun Chang, Fang Li

**Affiliations:** 1 Shanxi Key Laboratory of Cervical Disease Research, Jincheng People’s Hospital, Jincheng, China; 2 Obstetrics and Gynecology Medical Center, Jincheng Hospital Affiliated to Changzhi Medical College, Jincheng People’s Hospital, Jincheng, China; 3 College of Biomedical Engineering, Chongqing Medical University, Chongqing, China; 4 School of Public Health, Chongqing Medical University, Chongqing, China; 5 Jincheng Hospital Affiliated to Changzhi Medical College, Jincheng People’s Hospital, Jincheng, China

**Keywords:** case report, cervical low-grade squamous intraepithelial lesion (LSIL), gynecologic oncology, high-intensity focused ultrasound (HIFU), image-guided ablation, non-invasive therapy

## Abstract

**Background:**

Low-grade squamous intraepithelial lesions (LSIL) of the cervix and vagina are generally low-risk with a high likelihood of spontaneous regression, for which conservative management is preferred. However, treatment becomes challenging when patients exhibit persistent symptoms and imaging findings suggestive of malignancy, despite repeated biopsies confirming LSIL. This report describes the use of high-intensity focused ultrasound (HIFU) and focused ultrasound as non-invasive therapeutic strategies for complex cervical and vaginal lesions under such clinicopathologic discordance.

**Case presentation:**

This report presents the case of a 55-year-old postmenopausal woman with a 3-year history of intermittent postcoital bleeding and a 6-month history of vulvar itching. Serial MRI and PET/CT showed increased metabolic activity in the cervix and vagina, suspicious for malignancy, while multiple biopsies confirmed LSIL. The patient sequentially underwent treatment with HIFU and focused ultrasound using two different ultrasound therapeutic systems. Both procedures were well tolerated under conscious sedation, without intraoperative or postoperative complications. Symptoms improved markedly after the first treatment, and MRI confirmed effective ablation; focused ultrasound was subsequently applied to residual lesions. During clinical follow-up through 20 months, the patient remained symptom-free, and imaging, colposcopy, and targeted histopathology demonstrated regression of the cervical and vaginal lesions, with no visible evidence of recurrence. Baseline qualitative HPV DNA genotyping detected HPV56, whereas HPV16 and HPV18 were not detected. Extended HPV surveillance through May 2026 showed fluctuating genotype-specific results: HPV56 was no longer detected at the latest assessment, whereas HPV39 was intermittently detected. Sustained clearance of all high-risk HPV types was not demonstrated.

**Conclusion:**

In this selected patient with clinicopathologic discordance, sequential HIFU and focused ultrasound achieved symptom relief and complete lesion regressionthrough 20 months of follow-up, supporting that this approach may represent a potential option for patients with complex cervical and vaginal lesions who are not candidates for or decline conventional surgical intervention.

## Introduction

Low-grade squamous intraepithelial lesions (LSIL) of the cervix and vagina are generally managed conservatively due to their low malignant potential and high rates of spontaneous regression (1–3). However, persistent symptoms and imaging findings suspicious for malignancy may create diagnostic and therapeutic challenges, particularly when repeated biopsies fail to confirm high-grade lesions or carcinoma.

Therapeutic ultrasound is a rapidly evolving field with expanding applications across multiple medical specialties. High-intensity focused ultrasound (HIFU) and focused ultrasound are emerging non-invasive therapeutic modalities that have been successfully applied in gynecologic conditions such as uterine fibroids and adenomyosis, and have also been explored in selected cervical lesions (4, 5). A comprehensive 2026 review by Li et al. provides an updated overview of focused ultrasound surgery for cervical lesions, summarizing current evidence and emerging applications (6). Additionally, recent work by Dubernard et al. (2026) on HIFU for rectal endometriosis further illustrates the broader trend of expanding therapeutic ultrasound applications in pelvic diseases (7). These techniques enable precise tissue ablation while preserving surrounding structures, offering a potential alternative for patients who are poor candidates for surgery or who wish to avoid radical procedures.

It is important to clarify the terminology used in this report. HIFU (high-intensity focused ultrasound) is a subset of focused ultrasound that operates at higher intensity levels to achieve thermal coagulation necrosis. In this case, we used two different systems: the JC300 HIFU system (higher intensity, deeper penetration) and the CZF300 superficial focused ultrasound system (lower intensity, more superficial targeting). Both are forms of focused ultrasound but operate at different intensity levels and tissue depths.

Herein, we report the case of a 55-year-old woman with persistent postcoital bleeding and vulvar itching whose biopsies consistently demonstrated LSIL, whereas imaging studies raised suspicion for malignancy. Given this clinicopathologic discordance and the limitations of conventional treatment options, the patient underwent sequential high-intensity focused ultrasound (HIFU) and focused ultrasound for the cervical and vaginal lesions. This case highlights the potential value of sequential ultrasound-based therapy as an individualized second-line strategy under extreme anatomical constraints, offering a safe and effective non-invasive alternative in carefully selected patients who are not candidates for standard surgical management.

## Case presentation

### Patient information and clinical presentation

A 55-year-old postmenopausal woman, gravida 6 para 4, presented to our hospital on July 15, 2024, with a 3-year history of intermittent postcoital bleeding and a 6-month history of vulvar itching. She had undergone natural menopause at the age of 53 years. Her medical history was unremarkable, and her family history was notable for esophageal cancer in her mother.

### Diagnostic assessment

Clinical examination revealed hypopigmentation in the external genitalia, consistent with atrophic changes. Vaginal examination revealed indurated tissue in the upper segment of the vagina with limited elasticity. The cervix appeared hard in consistency, poorly visualized, and showed no contact bleeding (**Figure 1a**).

**Figure 1:**
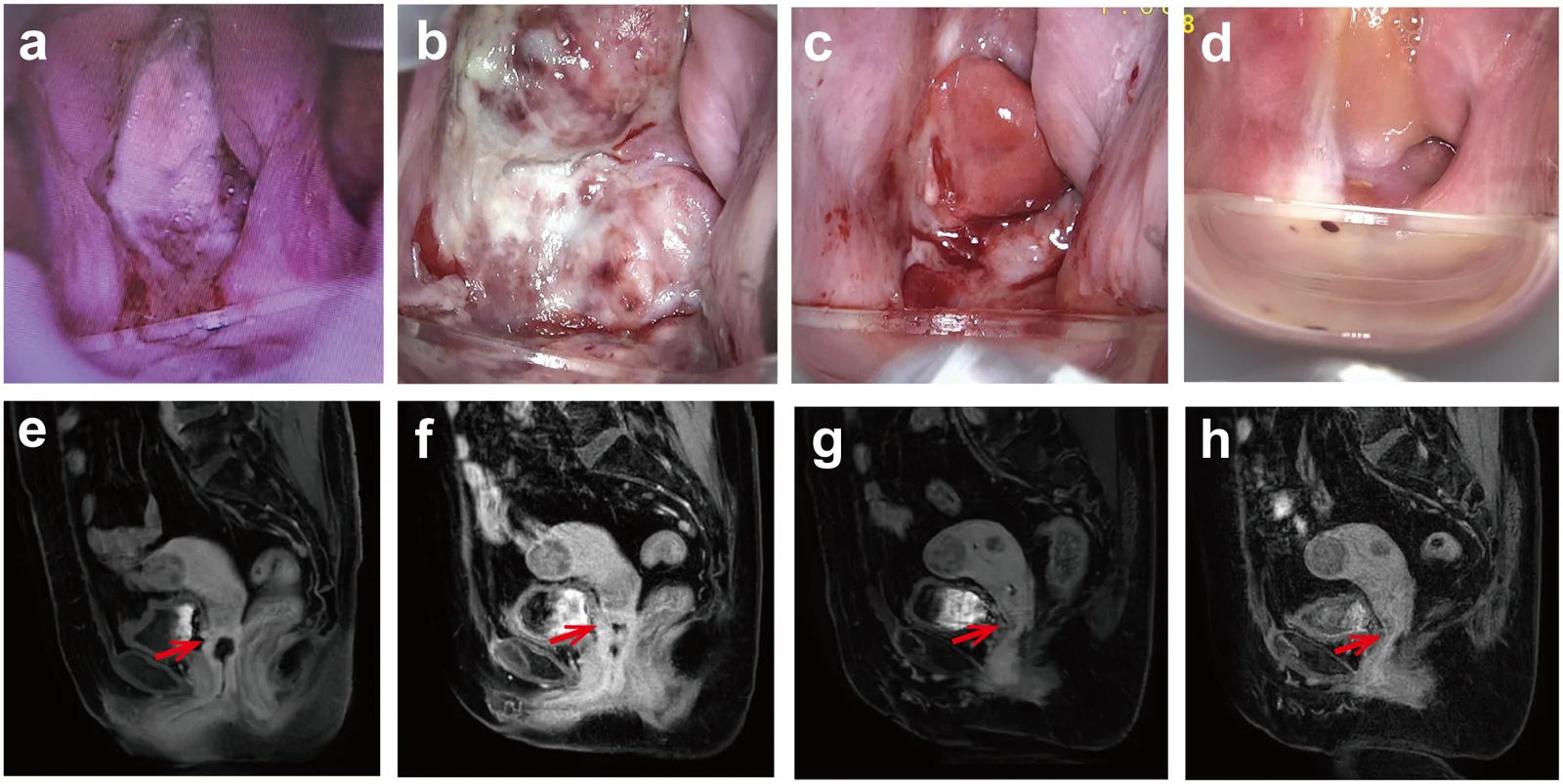
Colposcopic findings and sagittal magnetic resonance imaging (MRI) before and after high-intensity focused ultrasound (HIFU) treatment of cervical lesions. **(a–d)** Colposcopic views of the cervix and vagina before and after HIFU treatment. **(a)** Before treatment, the cervix was poorly exposed with a firm texture. A large whitish lesion with patchy distribution was observed on the surface, showing indistinct boundaries with the adjacent vaginal wall. **(b)** At 3 months after HIFU, the cervix became clearly exposed, with patchy whitish areas and scattered hemorrhagic foci visible on the surface. **(c)** At 7 months, the cervix was well exposed with a smooth and evenly colored surface, and a few scattered congested areas were noted locally. **(d)** At 20 months, the cervix appeared well exposed and soft in texture, with a smooth, uniformly colored surface and mild physiological congestion. **(e–h)** Sagittal contrast-enhanced T1-weighted MRI images of the pelvis. **(e)** Pre-treatment image shows a hyperintense cervical lesion with irregular morphology, the arrow indicates the cervical lesion. **(f)** At 3 months after HIFU ablation, the lesion demonstrates a marked reduction in both size and enhancement, the arrow indicates the residual lesion at the treated cervical site. **(g)** At 7 months, the lesion had completely resolved, with no residual abnormal enhancement; the arrow indicates the previous lesion site after HIFU treatment. **(h)** At 16 months, complete radiological resolution was maintained, with no recurrent or residual abnormal enhancement; the arrow indicates the treated cervical region corresponding to the original lesion site.

Imaging studies, including pelvic MRI and PET/CT, demonstrated progressive abnormalities in the cervix and vagina. PET/CT was ordered because of the highly suspicious MRI findings (enhancing cervical mass with irregular borders) in a postmenopausal woman with bleeding. The gynecologic oncology team requested PET/CT to evaluate for metastatic disease and rule out invasive cancer before planning management. PET/CT showed increased metabolic activity in the cervix and upper vagina, raising suspicion for malignancy (**Figures 1E**, **2**).

**Figure 2:**
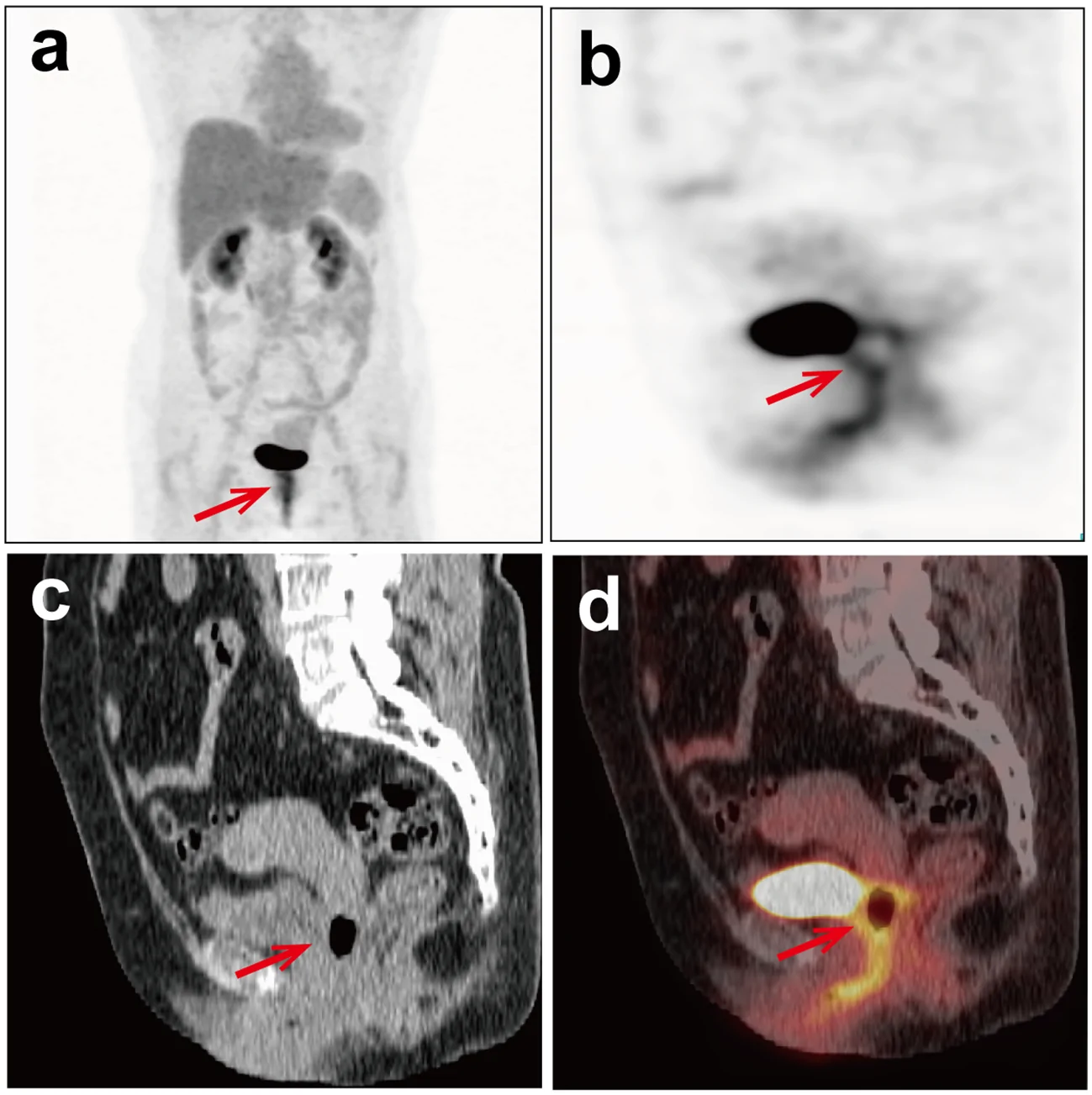
Pre-treatment fluorodeoxyglucose positron emission tomography/computed tomography (FDG PET/CT) findings of the cervical lesion.All arrows indicate the location of the target cervical lesion. **(a)** Whole-body maximum-intensity projection (MIP) PET image shows a focal area of markedly increased FDG uptake in the pelvis; the arrow indicates the hypermetabolic focus corresponding to the cervical lesion. No other definite abnormal hypermetabolic focus suggestive of distant metastasis is identified. **(b)** Sagittal PET image demonstrates intense focal FDG uptake in the cervical region; the arrow indicates the metabolically active cervical lesion. **(c)** Corresponding sagittal non-contrast CT image shows the anatomical location of the cervical abnormality; the arrow indicates the cervical lesion/site corresponding to the hypermetabolic focus on PET. **(d)** Sagittal fused PET/CT image confirms localization of the intense FDG uptake to the cervix; the arrow indicates the hypermetabolic cervical lesion precisely localized on the fused image. The marked FDG avidity raised strong suspicion of malignancy despite the benign findings on biopsy.

However, repeated colposcopy-directed biopsies were performed at different time points. In February 2021, two specimens were obtained from the upper anterior vaginal wall. During the immediate pretreatment assessment in July 2024 at Jincheng People’s Hospital, four specimens were obtained, including two cervical biopsies from the 6- and 9-o’clock positions and two biopsies from the upper anterior vaginal wall. In August 2024, two additional vaginal biopsy specimens were obtained at Peking Union Medical College Hospital; these specimens consisted predominantly of necrotic material with focal calcification and lacked sufficient viable squamous epithelium for definitive grading. Meanwhile, the previously obtained vaginal biopsy slides were reviewed through expert pathological consultation at Peking Union Medical College Hospital, and the consultation diagnosis was vaginal LSIL. P16 and Ki-67 immunohistochemistry were performed on the pretreatment cervical and vaginal specimens containing evaluable viable epithelium. Focal and patchy, non-block-type p16 positivity and Ki-67 staining predominantly confined to the lower third of the epithelium supported the diagnosis of LSIL (**Figures 3a–d**). No histopathological evidence of HSIL or invasive carcinoma was identified in the sampled viable tissues.

**Figure 3:**
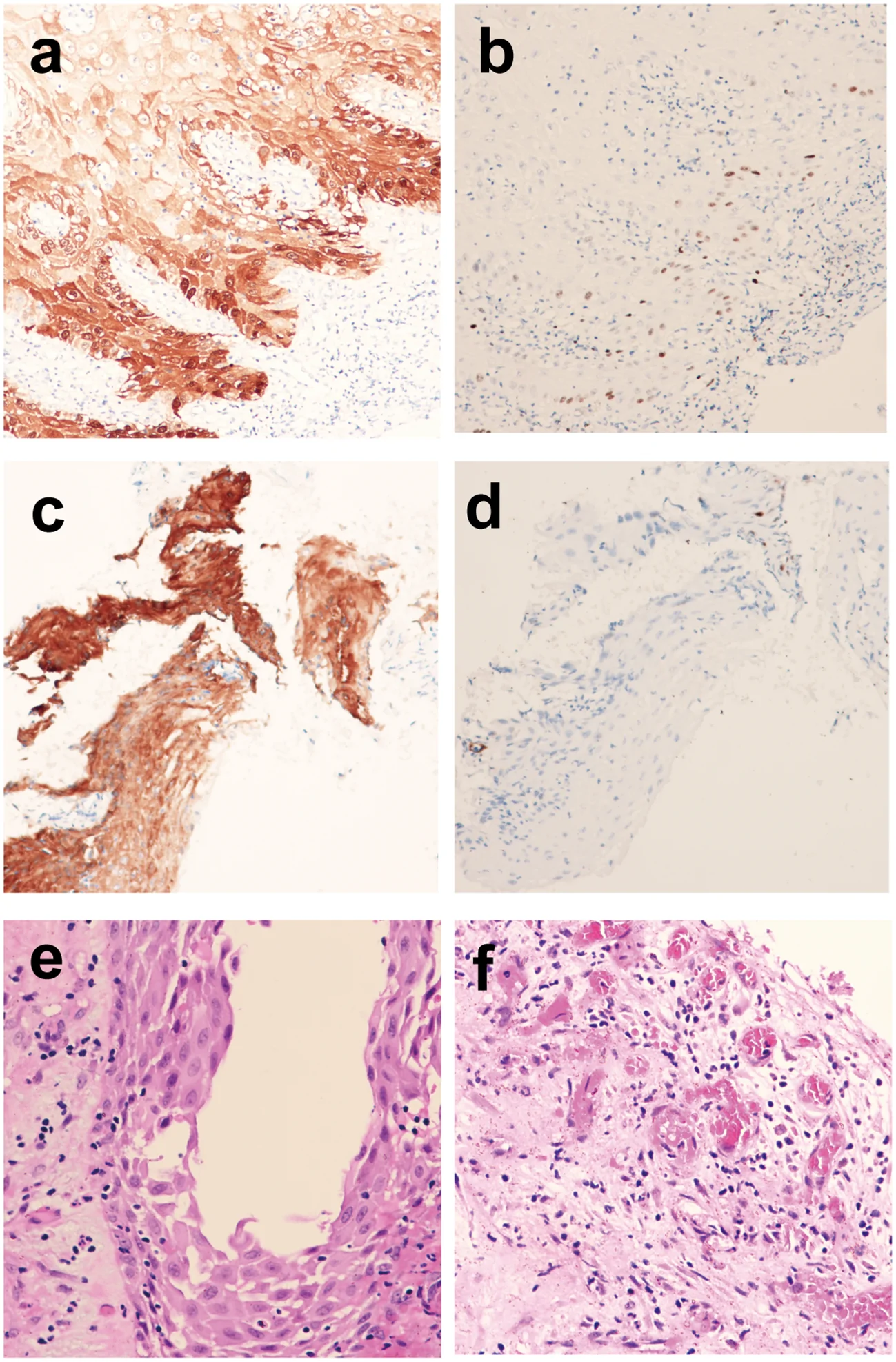
Immunohistochemical examination of pretreatment cervical and vaginal wall biopsy specimens and histopathological findings at the 7-month follow-up after the first HIFU treatment. **(a)** Cervical biopsy specimen showing focal and patchy, non-block-type p16 immunoreactivity (×100). **(b)** Ki-67 immunostaining in the cervical biopsy specimen, predominantly confined to the lower third of the squamous epithelium (×100). **(c)** Vaginal wall biopsy specimen showing focal p16 immunoreactivity (×100). **(d)** Ki-67 immunostaining in the vaginal wall biopsy specimen, confined to the lower third of the squamous epithelium (×100). **(e)** Hematoxylin and eosin (H&E) staining of the vaginal wall biopsy specimen at the 7-month follow-up shows intact squamous epithelium with mature differentiation and no evidence of intraepithelial neoplasia or residual lesion (×200). **(f)** H&E staining of the cervical biopsy specimen at the 7-month follow-up shows intact squamous epithelium with mature differentiation and no evidence of intraepithelial neoplasia or residual lesion (×200).

Endocervical curettage (ECC) was not feasible due to severe cervical anatomical distortion, which obscured the external os and prevented adequate exposure. To evaluate deep cervical tissue and rule out occult invasion, an ultrasound-guided interstitial core needle biopsy of the cervix was performed; histology showed only chronic inflammation without evidence of invasive malignancy.

Qualitative PCR-based HPV DNA genotyping performed in July 2024, before HIFU treatment, detected HPV56, whereas HPV16 and HPV18 were not detected. The assay provided qualitative genotype-specific results and did not quantify viral load.

This marked discordance between the progressive imaging findings and the pathological results, together with the patient’s persistent symptoms, posed a significant diagnostic and therapeutic challenge.

### Therapeutic intervention

Given the persistent symptoms, the marked discrepancy between imaging and pathology, and after multidisciplinary discussion with the gynecologic oncology, radiology, and pathology teams, sequential focused ultrasound therapy was selected as a non-invasive treatment option. The rationale for this approach and the device specifications are detailed below.

### Device specifications

Two different focused ultrasound systems were used sequentially in this case, each with distinct characteristics:

JC300: A high-intensity focused ultrasound system that induces thermal coagulation necrosis with a focal intensity above 1000 W/cm². Operating at 1.0 MHz with a maximum output power of 400 W, it features deep tissue penetration and targets lesions 1–15 cm in depth, making it suitable for larger-volume lesions. It was used for the first treatment session to cover potential occult deep involvement given radiologic suspicion and clinicopathologic discordance. Treatment parameters were adapted from standard uterine fibroid protocols with downward-adjusted focal depth for cervical targeting. A 5–8 mm perilesional safety margin was maintained, with real-time ultrasound monitoring to keep energy delivery at least 10 mm away from the bladder and rectal mucosa.

CZF300: A superficial focused ultrasound system that delivers mild hyperthermia and mechanical effects to trigger cellular apoptosis and tissue repair, with a focal intensity of 100–300 W/cm² and no frank coagulation necrosis. Operating at 10 MHz (10-fold the JC300 frequency) with a maximum output power of 8.4 W, it offers finer focal resolution for superficial mucosal lesions 2–10 mm in depth. It was applied in the second session to precisely target residual superficial LSIL after initial deep ablation, preventing overtreatment and stromal damage to thin postmenopausal atrophic epithelium. Parameters were modified from cervical intraepithelial neoplasia protocols, with ~15% energy reduction for atrophic mucosa and a 1–3 mm stromal safety margin. The fractionated two-session regimen allowed intermediate tissue recovery and reduced adverse event risk from the large treatment volume.

### First treatment (HIFU)

On September 11, 2024, the patient underwent HIFU treatment using the JC 300 Focused Ultrasound Tumor Therapeutic System (Chongqing Haifu Medical Technology Co., Ltd., Chongqing, China) at a sonication power of 400 W. A significant grayscale change was observed after 36 s of sonication, and treatment was terminated once the grayscale change covered the entire targeted area. The total sonication time was 303 s. The procedure was well tolerated under conscious sedation, with only mild pain (pain score <3). The patient experienced no activity restrictions and no treatment-related complications were observed.

### Second treatment (focused ultrasound)

Because residual lesions were still detected on colposcopy at the 3-month follow-up, the patient underwent superficial focused ultrasound treatment on January 6, 2025. Residual lesions were treated using the small probe of the CZF300 Ultrasonic Gynecological Therapeutic System (Chongqing Haifu Medical Technology Co., Ltd., Chongqing, China). The treatment power was 8.4 W (level 3), the operating frequency was 10 MHz, the pulse frequency was 1000 Hz, the cumulative treatment time was 295 s, and the cumulative energy delivered was 877 J. The procedure was well tolerated, and no complications were observed.

### Follow-up and outcomes

The patient was followed up regularly. At the 1-month follow-up, her symptoms, including vaginal bleeding and vulvar itching, had markedly improved. At the 3-month follow-up, pelvic MRI showed further reduction of the lesions (**Figure 1f**). However, colposcopic examination revealed residual lesions in the cervix and vagina (**Figure 1b**).

By 7 months after the initial HIFU treatment, the patient remained symptom-free, and both colposcopy and imaging demonstrated complete regression of cervical and vaginal lesions (**Figures 1c, g**), targeted biopsies of the cervix (6-o’clock position) and upper anterior vaginal wall were obtained, and histopathologic examination further confirmed the absence of residual disease (**Figures 3e, f**). At 16 months, pelvic MRI continued to show sustained complete regression of all lesions (**Figure 1h**). At the 20-month follow-up, colposcopy showed complete epithelial healing and normalization of the cervix and vagina, with no evidence of recurrence (**Figure 1d**).

Qualitative HPV DNA genotyping was repeated during follow-up. On April 30, 2025, both HPV56 and HPV39 were detected. By 16 months after the initial HIFU treatment, HPV56 remained detectable, whereas HPV39 was not detected. At the latest assessment, approximately 20 months after the initial HIFU treatment, HPV56 was no longer detected, whereas HPV39 was again detected. The timeline of the patient’s clinical course is shown in **Figure 4**, which depicts the key events from initial presentation through diagnosis, treatment, and follow-up, including the dates of biopsy procedures, both treatment sessions, and follow-up visits with corresponding clinical and imaging findings.

**Figure 4:**
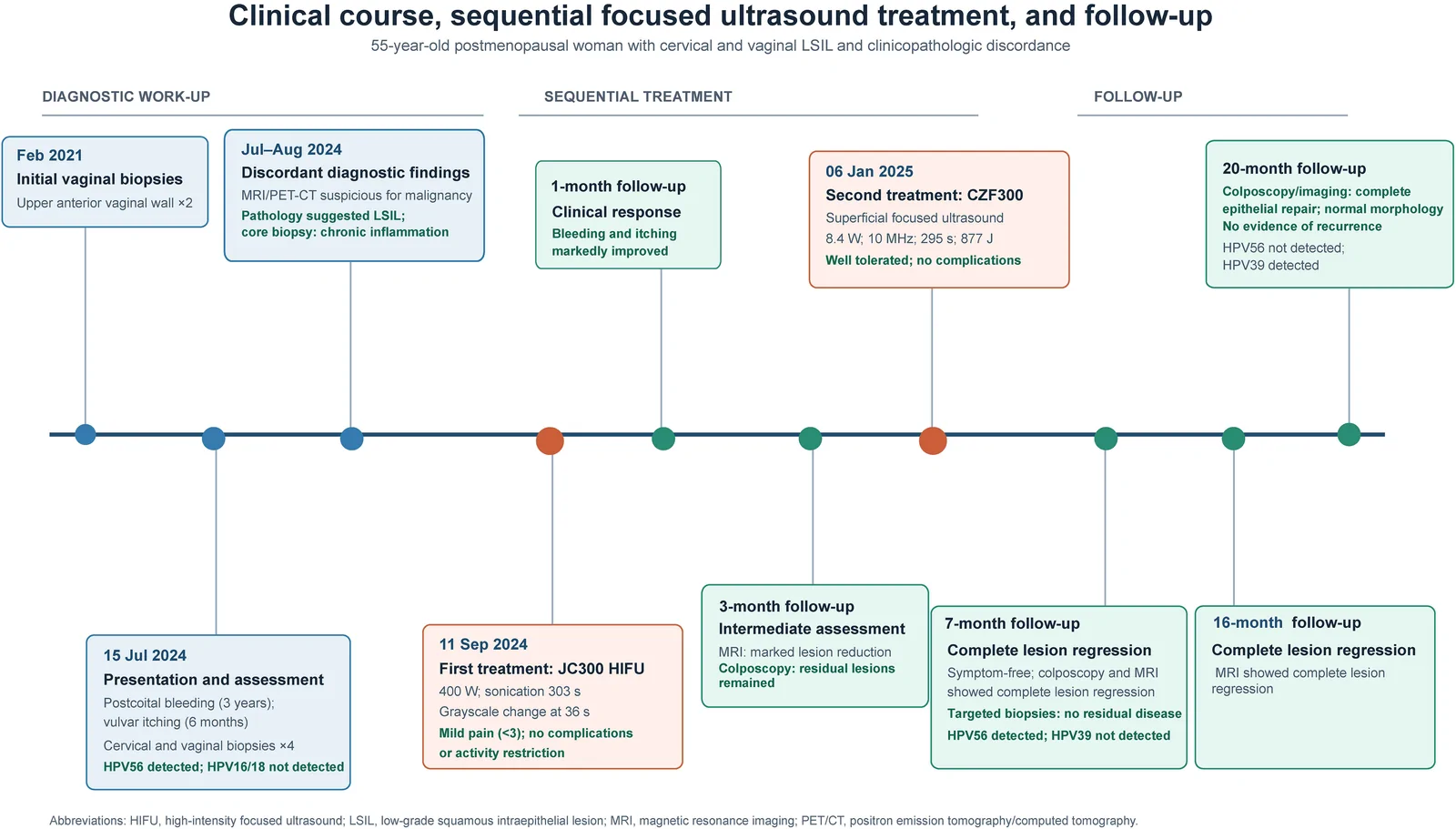
Time-event axis.

## Discussion

Cervical and vaginal LSIL are clinically relevant entities in gynecological practice. Cervical LSIL (CIN1) is a common histologic diagnosis (8), whereas vaginal LSIL (VaIN1) is comparatively uncommon (9). According to current guidelines and published studies, LSIL is generally characterized by a low risk of progression and a high likelihood of spontaneous regression; therefore, conservative management is usually recommended (9–11). Nevertheless, clinical management becomes challenging when persistent symptoms are present or when imaging findings raise suspicion for malignancy, while repeated biopsies fail to demonstrate high-grade lesions or invasive disease.

In the present case, a 55-year-old postmenopausal woman presented with a 3-year history of intermittent postcoital bleeding and a 6-month history of vulvar itching. Pelvic MRI and PET/CT demonstrated progressive abnormalities in the cervix and vagina, with findings highly suspicious for malignancy. However, repeated biopsies revealed only chronic inflammation initially and later low-grade squamous intraepithelial lesions (LSIL) involving both the cervix and vagina. This marked clinicopathologic discordance posed a significant diagnostic and therapeutic dilemma.

Postmenopausal atrophy and associated inflammation may have contributed to this clinicopathologic discordance. Estrogen-deficient vulvovaginal mucosa becomes thin and friable and may cause postcoital bleeding (12). Coexisting cervical inflammation can produce cervical enlargement and abnormal enhancement or hyperemia on CT or MRI, while benign inflammatory processes may also demonstrate increased FDG uptake and result in false-positive PET/CT findings (13, 14). These mechanisms may partly explain why the imaging findings appeared disproportionately suspicious relative to the histopathological findings. However, neither atrophy nor inflammation can establish or exclude malignancy. Accordingly, repeated histopathological assessment remained essential, and the possibility of unsampled endocervical or invasive disease could not be completely excluded.

Observation would ordinarily have been appropriate for histologically confirmed LSIL. In standard gynecologic oncology practice, however, persistent postcoital bleeding combined with a non-visualized squamocolumnar junction (SCJ) and FDG-avid pelvic imaging typically warrants definitive surgical excision to rule out occult invasive disease. The two standard-of-care pathways are diagnostic conization/loop electrosurgical excision procedure (LEEP) for cervical-localized lesions, and simple (Querleu-Morrow Type A) hysterectomy with intraoperative frozen section assessment for cases with broader vaginal extension or high suspicion of invasion.

In this case, neither surgical pathway was considered technically feasible or clinically optimal for three key reasons. First, severe cervical stenosis and anatomical distortion completely obscured the external os and prevented adequate visualization of the transformation zone, making it technically impossible to obtain a complete, representative excisional specimen via conization or LEEP; an incomplete excision would carry a high risk of missed deep or endocervical disease. Second, the lesion extended continuously to the upper vaginal wall, which would not be adequately addressed by cervical conization alone, and hysterectomy would have been required for full extirpation. Third, after multidisciplinary review of all pathological samples (including repeated colposcopic biopsies and ultrasound-guided core needle biopsy), the estimated risk of occult invasive carcinoma was deemed low in the context of confirmed LSIL on all evaluable specimens. Following detailed discussion of all options, including the risks and benefits of surgical excision versus non-invasive ablation, the patient declined invasive surgical procedures and elected an individualized non-surgical strategy with close post-treatment surveillance.

Conventional management for cervical and vaginal lesions suspicious for high-grade disease or malignancy often involves surgical intervention, such as conization, local lesion excision, or hysterectomy, depending on the extent and nature of the lesion. Although effective, these approaches are invasive and may be associated with operative complications, tissue scarring, prolonged recovery, and, in younger patients, loss of fertility (15, 16).

As a non-invasive treatment, HIFU has been widely used to treat different types of solid tumors (17–20). Performed under ultrasound guidance, HIFU enables precise and selective ablation of target lesions (21). HIFU causes coagulation necrosis of the target mass lesion without damaging to the surrounding tissues that do not want to be treated (22).

In recent years, HIFU and focused ultrasound have also been explored in the treatment of cervical and vaginal lesions. In a retrospective study of 153 patients with residual or recurrent cervical cancer after chemoradiotherapy, HIFU achieved a disease control rate of 93.5% with an acceptable safety profile (23). In women desiring fertility preservation and diagnosed with CIN2/3, HIFU combined with antiviral therapy has shown promising efficacy, with reported cure rates of 82.8% at 7 months and 96.6% within 1 year (24). Focused ultrasound has also demonstrated favorable efficacy in CIN1, with good tolerability, no severe complications, and encouraging lesion regression and HPV clearance (25).

Regarding vaginal intraepithelial neoplasia (VaIN), the evidence base for HIFU treatment is very limited, consisting primarily of small case series from a few centers. Wu et al. reported promising results in a small series, but larger studies are needed to confirm these findings (26). Our case adds to this emerging but preliminary evidence base.

The combined application of HIFU and focused ultrasound in the treatment of such complex cervical and vaginal lesions has rarely been reported. In our case, the sequential approach was chosen based on the lesion characteristics and the complementary strengths of the two devices. The initial HIFU treatment addressed the deeper and larger volume component of the lesion, while the subsequent superficial focused ultrasound treatment targeted the residual mucosal disease with greater precision. This sequential approach allowed for comprehensive treatment of both deep and superficial components while minimizing the risk of adverse effects.

In our case, the patient tolerated both procedures well, and no intraoperative or postoperative complications were observed. During follow-up, the patient remained free of the presenting symptoms, and the clinical, imaging, colposcopic, and histopathological assessments performed during the first 12 months showed regression of the cervical and vaginal lesions. Extended HPV surveillance through approximately 20 months demonstrated fluctuating genotype-specific detection rather than sustained clearance of all high-risk HPV types. Imaging and histopathologic evaluations confirmed complete regression of the lesions, suggesting that sequential HIFU and superficial focused ultrasound may represent an individualized second-line therapeutic option under extreme anatomical constraints for carefully selected patients with cervical and vaginal intraepithelial lesions in whom conventional surgical approaches are infeasible or declined.

Several important limitations of this case should be acknowledged: Ablation without definitive histologic diagnosis may mask underlying malignancy; this case was performed only after multiple negative biopsies, negative ultrasound-guided core needle biopsy, multidisciplinary discussion, and informed consent with close follow-up, representing a selected individualized scenario rather than routine practice. The 20-month follow-up is insufficient to confirm long-term treatment durability and exclude late recurrence. Moreover, as a single-center case report amid sparse evidence for HIFU in vaginal intraepithelial neoplasia, its generalizability is limited. Larger multicenter studies with longer follow-up are warranted to verify these findings and define the clinical role of this approach.

## Conclusions

In this case, sequential HIFU and superficial focused ultrasound achieved marked symptom relief and complete regression of cervical and vaginal LSIL without treatment-related complications at 20-month follow-up. These findings suggest that ultrasound-based non-invasive therapy may be serve as an individualized second-line strategy under severe anatomical constraints for carefully selected patients with cervical and vaginal intraepithelial lesions in the setting of clinicopathologic discordance, particularly when standard surgical approaches are infeasible or declined by the patient. However, given the limitations of a single case report and short follow-up duration, further studies with larger sample sizes and longer follow-up are warranted to confirm these findings and establish the role of this approach in clinical practice.

## Data Availability

The raw data supporting the conclusions of this article will be made available by the authors, without undue reservation.
